# Normal Physiologic Birth Continuing Professional Development: From a National Health Priority to Expanded Capacity

**DOI:** 10.5334/aogh.3247

**Published:** 2021-10-08

**Authors:** John K. Shakpeh, Mary W. Tiah, Cecelia C. Kpangbala-Flomo, Rita Florence Matte, Sodey C. Lake, Susan D. Altman, Tanya Tringali, Kerry Stalonas, Lloyd Goldsamt, Lily Zogbaum, Robin Toft Klar

**Affiliations:** 1 Redemption Hospital, Ministry of Health, Republic of Liberia, LR; 2 Liberian Board for Nursing and Midwifery, LR; 3 New York University Rory Meyers College of Nursing, US

## Abstract

**Background:**

The Republic of Liberia has experienced many barriers to maintaining the quality of its healthcare workforce. The Resilient and Responsive Health Systems (RRHS) Initiative supported by the U.S. President’s Emergency Plan for AIDS Relief (PEPFAR) has responded to Liberian identified health priorities. Liberia’s maternal morbidity and mortality rates continue to rank among the highest in the world. Recent country regulations have put forth required continuing professional development (CPD) for all licensed healthcare workers for re-licensure.

**Methods:**

The Model for Improvement was the guiding framework for this CPD to improve midwifery and nursing competencies in assisting birthing women. Two novel activities were used in the CPD. We tested the formal CPD application and approval process as this is a recent regulatory body policy. We also included the use of simulation and its processes as a pedagogical method. Over a two-year period, we developed a two-day CPD module, using didactic training and clinical simulation, for Liberian midwives. We then piloted the module in Liberia, training a group of 21 participants, including midwives and nurses, including pre- and post-test surveys as well as observational evaluation of participant skills.

**Findings:**

There were no significant changes in knowledge acquisition noted in the post-test. Small tests of change were implemented during the program, supporting the stages of the Model of Improvement. Observation of skill acquisition was done; however, using a formal observation checklist, such as an Observed Structured Clinical Evaluation (OSCE), would add more robust findings. The CPD and follow-up activity highlighted the need for human and financial support to maintain the simulation kits and to create sustainability for future trainings. Videotaping the didactic and simulation two-day continuing professional development train-the-trainer workshop expands the sustainability beyond newly prepared trainers. Simultaneous with this CPD, the Liberian Board for Nursing and Midwifery (LBNM) worked with a partner to create a CPD portal. The CPD partners created modules from the videos and have uploaded these modules to the LBNM’s new CPD portal.

**Conclusions:**

Using a quality improvement model as a framework for developing and implementing CPDs provides a clear structure and supports the dynamic interactions in learning and clinical care. It is too soon to determine measurable health outcomes resulting from this project. Anecdotal feedback from clinicians and leaders was not directly related to the content of the CPD; however, it does demonstrate an increased awareness of examining changes in practice to support expanded health outcomes. Further research to examine methods and processes to determine the quality and safety outcomes of CPD trainings is necessary.

## Background

Healthcare professional education does not end upon graduation. Evidence-based practice is updated constantly, and it is expected that healthcare providers will stay up to date to provide the highest quality care. Not only do healthcare providers need to know the science of the evolving evidence, but also they need to demonstrate the related competencies to support their care [1].

The Republic of Liberia has experienced many barriers to maintaining the initial education and continuing professional development (CPD) of healthcare providers. Two civil wars and the Ebola epidemic created enormous gaps in education and CPD. Since 2014, the Ministries of Health and Finance have developed a strategic plan to increase the quantity and quality of healthcare providers in Liberia [2].

In 2016 a regulatory policy was initiated to require all licensed health professionals in Liberia to demonstrate profession-dependent credit hours earned to become relicensed to practice. The Liberian regulatory bodies released the Standard Operating Procedure (SOP) for CPD for Liberia Health Professionals with the purpose to ensure continuous professional competency as a way of improving health outcomes and as a pre-requisite for maintaining professional licensure in Liberia [3]. Some of the key principles behind a CPD maintain that it should address priority health concerns, build upon professionals’ existing knowledge and experience, link their learning to practice and therefore be relevant to their current and future practice, and be provided in an environment conducive to effective learning [4,5].

Liberia is one of the African countries south of the Sahara that still has a high maternal mortality rate. According to UNICEF, Liberia has one of the highest Maternal Mortality Rates in the world: 1,072 deaths for every 100,000 births [6]. The mortality rate of newborns, within the first 28 days of life, is equally high: 37 for every 1,000 live births. One of the gaps that was identified by the International Midwifery Conference (IMC) /United Nations Fund for Population Activities (UNFPA) in their 2012 analysis was Liberia’s lack of high-quality, competency-based midwifery education programs and a shortage of competent midwifery trainers. Furthermore, according to the State of the World’s Midwifery Report in 2011, providing midwives with the correct training, equipment, and support could decrease the rate of maternal, fetal, and newborn deaths by 56% [7]. This is because the quality of those trainers, in terms of skills acquisition and competence, directly determines the level of performance of health service delivery.

The Ministry of Health reports that the leading cause of maternal death in Liberia is postpartum hemorrhage [7]. Therefore it is important that when developing CPDs they are designed to train midwives and nurses to focus on this serious maternal complication. Furthermore, the CPD training modules should be developed in order to maintain up-to-date skills and knowledge concerning protocols, guidelines, and safe practice.

## Development of Normal Physiologic Birth Train-the-Trainer for CPD

### Framework

The CPD was developed using the Model for Improvement as a guiding framework. The Institute for Healthcare Improvement identified the steps of the Model for Improvement: 1.) Introduction, 2.) Forming the team, 3.) Setting the aims, 4.) Evaluating measures, 5.) Selecting changes, 6.) Tracking changes, 7.) Implementing changes, and 8.) Spreading changes [8].

### Introduction/Forming the Team

Knowing the background of midwifery education and contextual influences on midwifery care over the past three decades, we expanded an already existing partnership to support the development of this CPD for midwives and nurses in Liberia to support quality and competence in the care of women and newborns. This was a quality improvement project; therefore, institutional review board review was not required. Members of the multilateral partnership included representatives from Liberia and the United States (USA). In Liberia, the Chief Nurse at Liberia’s largest tertiary care public hospital and the Liberian Board for Nursing and Midwifery (LBNM) Director of Nursing and Midwifery Programs and Registrar were our critical partners. In the USA, the academic institutional partner expertise included midwifery faculty, international health services researcher/principal investigator, monitoring and evaluation, and international operations experts.

### Setting Aims

At the time that this CPD was being developed, the Essential Competencies for Midwifery Practice [9] had not yet been released. However, a 2018 update [10] provided insight into the upcoming changes, which served as a foundation for the development of this first CPD. These updated guidelines emphasize normal physiologic birth, evidence-based practice, reduction of unnecessary interventions, the midwife’s autonomy, and the role of the midwife in emergency management. Additionally, respectful, patient-centered care principles, such as shared decision making, informed consent, and promotion of human rights, were highlighted in the revision and are well aligned with the educational and clinical priorities of the LBNM. During initial discussions by stakeholders about topics to be considered for the introductory CPD, those of normal physiologic birth concepts, which the participants were familiar with, was chosen for the initial simulation in order to orient them to the simulation pedagogy. This allowed for transference of simulation skills into the more complex simulation on PPH that was to follow. The Essential Competencies for Midwifery Practice 2018 Update [10] outlines teaching pedagogy, such as in-person simulation, which supports midwifery student learning using all domains such as visual, psychomotor, and auditory. Expanding simulation as a pedagogy to support competencies beyond students to health care providers has demonstrated value [11] and is supported by the Institute of Medicine’s redesign of continuing education for health professionals [5]

The Training of Trainers (ToT) model is designed to engage master trainers in the teaching of less experienced participants with the goal of increasing the limited pool of qualified instructors [5] available in Liberia. Having new participants watch experienced trainers teach this method makes it possible for one expert to train many others, which allows for increased dissemination of content to all areas of the country.

Additionally, the ProntoPack, a birthing simulation training kit, was selected for use in this CPD because it is portable, affordable, low tech, yet high fidelity, making it an appropriate option for low resource, high need countries such as Liberia. As of 2019, Pronto International has trained over 7,000 providers in 13 countries. Research has shown [11] the ProntoPack and the accompanying SimPacks, which provide simulation prompts/scripts, to be a helpful tool that can be used to increase rates of respectful care, improved team communication, and management of obstetrical emergencies, such as PPH [7] Eight ProntoPacks were purchased and transported to Liberia for use in this project with the intention that they would be used in the future for other life threatening obstetrical complications, such as preeclampsia and obstructed/prolonged labor.

### Establishing Measures

After the didactic content and teaching/learning methods were finalized, the U.S. academic institutional partner’s Clinical Simulation Learning Center (CSLC), with its facilities and highly qualified staff, as well as midwifery student volunteers, were used to test the simulations. One goal of this testing was to determine the necessary equipment needed to produce a high-fidelity simulation that could be easily reproduced.

### Selecting Changes

The testing revealed that a number of supplies would be needed to complete the simulation, for this low resource country, which are not commonly found in high-tech simulation centers, such as funnels to fill bags with simulated blood and dental floss to tie off umbilical cords. These supplies were purchased, or donated by the CSLC. In the future, these additional items will be replaced with relative ease and at a low cost. Another goal of the test simulations was to make sure that the U.S. academic partners were familiar with how the equipment worked and that it worked properly. Through some repetition, this was accomplished.

The entire team identified the importance of sustainability of this CPD. The intention of ToT initiatives is to create a layer of trainers who can then take the program to other areas of the country. Appreciating the geographic and infrastructure context of Liberia, we asked for and received financial support from our funder to hire a videographer to film the entire two-day training. Unbeknownst to all of us at the time, having the videos of this training proved to be even more forward-thinking than originally intended.

In Liberia, obstetrical nurses assist with births, although not as frequently as midwives; therefore, they were both included in the first cohort of participants. The Chief Nursing Officer of Redemption Hospital in Monrovia, within whose facility we had agreed to conduct the CPD, contacted the supervisors on the obstetrics/gynecology (OB/GYN) ward there along with supervisors of other institutions. A total of 9 public and private hospitals and health centers sent a total of 21 participants.

### Tracking Changes

**Figure 1:**
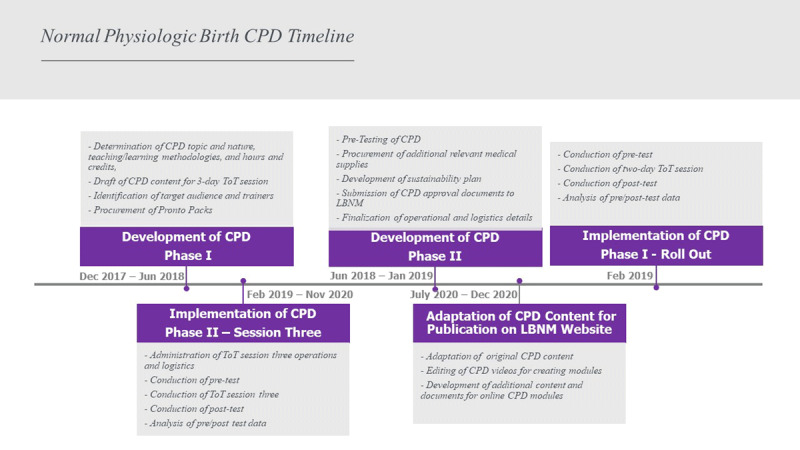
Timeline for Development and Implementation of Normal Physiologic Birth CPD.

The teamwork and coordination for development, planning, and implementation of this CPD was complex. Regular WhatsApp calls and emails concerning the planning and delivery of the program kept all of the moving parts in order. All timelines were met in the leadup to the CPD training in February 2019. Adding to the complexity was that these steps were iterative, requiring adaptations when reviewed by each of the partners. ***Table 1*** highlights the complexity, and ***Figure 1*** provides a timeline for this CPD.

**Table 1:** Steps in Development of Normal Physiologic Birth CPD.


Development and submission of the CPD Approval Form to the Liberian regulatory board, and CPD approval by the LBNM.

Procurement of pronto-packs and other supplies to complete the eight Simulation Packs to be left in-country

Invitation letters to 21 participants from 9 different institutions in the Montserrado County (the county surrounding Monrovia the capitol city), and agreement by the Directors of Nursing from all institutions for their participation

Finalization of all logistical arrangements including daily travel per diem for participants, catering for the two days, and arrangement of videographer for the event

Upon completion of the 20 hours of training, 20 credits to be awarded to participants to go towards their licensing renewal (Pending)

As the CPD was developed as a Training of Trainers framework, three participants were self-selected at the end of the two days to be CPD Leaders. These CPD leaders agreed to lead additional CPD trainings and to engage participants to lead trainings over time.


## Implementation of the Normal Physiologic Birth CPD Train-the-Trainer

### Implementing Changes

The entire team hit a major barrier as soon as the U.S. team arrived in Liberia for the implementation. Our original intention was to hold the two-day training at Redemption Hospital to serve as an exemplar for conducting CPD training within the clinical institution. However, the CPD was dependent on having electricity for running the projector and all of the filming equipment. We were notified that there was a shortage of power at Redemption, so we swiftly worked with other Liberian partners to arrange space. We all transitioned to the new venue and implemented the CPD training. The participants were excited to be there, spurred on by the clinical leader’s motivating presentation to start the program.

The session started with a pretest to identify the baseline level of knowledge of the group. Questions were derived from assigned articles and simulation activities.

In February 2019, the CPD was delivered as a two-day ToT session to 21 participants. The training took place in Monrovia, and 10 participants (46.6%) worked in hospitals in Monrovia, with the rest working throughout the country. Nearly all participants (19/21, 90.5%) were midwives (13, 61.9% were both nurses and midwives); only 2 (9.5%) were nurses who were not midwives. Participants had been practicing for an average of 11.4 years (range 1–23 year, SD = 5.9 years). Two thirds (14/21, 67.7%) had delivered a baby in the past month, with an average of 17.5 deliveries per participant (range 4–50 deliveries, SD = 15.8 deliveries).

Challenges are inevitable when teaching and learning about simulation. Despite having prior experience facilitating simulations with U.S. midwifery students, testing at the U.S. academic institution’s CSLC, as well as additional practice in Liberia, some technical difficulties were noted during the on-site training. Placement, management, and control of tubing for blood flow by the participant playing the birthing person proved to be more difficult than expected. However, these issues were not insurmountable and were remedied through repetition. These just-in-time repetitions during the simulation were micro tests of change, which demonstrated critical thinking from all participants [12] Another challenge emerged in relation to using debriefing skills, which is arguably one of the most important aspects of simulation, as it allows for improved learning and retention. Despite teaching this content in advance of the first simulation, this concept proved difficult for participants, as this was a new learning strategy for them. In future CPDs, providing educational content prior to the on-site training will allow the participants to be more familiar with this pedagogy. These challenges ultimately strengthened the impact of the train-the-trainer session, as they identified problems that could be remedied prior to actual implementation and also modeled the active problem-solving that is an essential part of these activities.

## Post CPD Implementation Activities

### Testing Changes

At the conclusion of the CPD, participants were asked to rate their experience using a series of questions answered on 5-point Likert scales. These questions included ratings of general aspects of the training (e.g., facilitators, materials; 10 questions, poor to excellent) and 14 questions assessing specific training areas (e.g., relevance to work, likelihood of using skills; 14 questions, not at all to very much). For the general satisfaction items, all average scores were greater than 4 (range 4.33 to 4.65) and individual items scores ranged from 3 to 5. For the specific items, all average scores were at least 4 (range 4.00 to 4.88) and individual item scores ranged from 1 to 5.

Participants were asked to indicate how much they had learned about normal birth delivery and postpartum hemorrhage as a result of the training. For each of these questions, 95.2% of participants (20/21) reported that they had learned some or a lot of new information. All participants reported that the training materials would be somewhat or very useful in their continued work and future trainings they will conduct.

Participant knowledge was assessed using a 15-item multiple choice survey administered prior to the start of the CPD and then readministered immediately following the conclusion. At pre-test, participants average score was 7.76 (range 6–10, SD = 1.26). At post-test, the scores averaged 8.47 (range 5–11, SD = 1.86). This difference was not statistically significant.

### Implementing Changes

We planned a follow-up for all 21 participants to demonstrate their simulation skills in serving as trainers for this CPD content. Due to time away from work limits, the follow-up was delayed. Additionally, the SARS-CoV-2 pandemic delayed even further the participant follow-up.

With a dedicated team, in November 2020, we successfully completed the follow-up activity face-to-face at Redemption Hospital. Of the 21 original participants, 19 responded that they would attend. Eleven participants attended and completed the session. The follow-up activity was led by three practicing midwives who participated in the original CPD training and self-identified as training champions.

After a recap of the original CPD ToT, each participant was given an opportunity to share how they had been disseminating their training experience. The sharing identified gaps and participants who needed additional support during the follow-up activity. The primary theme echoed the following participant’s response, “I had just graduated from college with inadequate skills and the training helped me a lot and I gained confidence in my midwifery practice.”

The Chief Nursing Officer of Redemption Hospital also attended the follow-up session. He noted that one of the participants shared that they had a very good experience because after training they had the experience especially with complicated deliveries. The midwife and supervisor are working together more.

Use of the partograph was not included in this CPD. The trainers included local objects in the training session to demonstrate cervical dilation. The participants were able to manipulate the objects to gain a clearer perception of each level of dilation. The majority of participants as well as facilitators appreciated the importance of observing cervical dilation for the commencement of the partograph and time for making the mother push.

### Spreading of changes

A follow-up of the CPD was conducted to support the participants’ confidence in training the next cadres of midwives and nurses. Due to workload commitments and the SARS-CoV 2 pandemic, there was a delay of over a year between the CPD training and this follow-up activity. This served as an opportunity to determine how the knowledge and skills learned in the CPD were retained past the end of the ToT.

The CPD champions, prior to delivering the post CPD activity, reviewed the simulation session overview and simulation materials. They got reacquainted to the simulation materials in the ProntoPack and set up the simulation area. Participants watched videos of normal birth of a vigorous baby and normal birth of a non-vigorous baby. All the participants took turns playing different roles (i.e., patient, nurse, midwife), which had been outlined to them as per the CPD guidelines. Each participant had to demonstrate how to assess the group for effective participation. After each simulation activity, there was a pre-brief and a debrief for the active participants and observers. These shorter activities provided further opportunities for small tests of change.

To determine how much knowledge had been retained from the CPD, this post CPD activity administered the same post-test used from the original CPD training. Eleven participants took the post-test. A score of 50% was considered passing. Six persons (55%) passed the test while five participants (45%) failed. Participants were returned their post-tests so that they could review the incorrect questions. These post-test scores highlight the need for continuing professional development along with in-service training conducted on a more regularly scheduled basis.

Additional spreading of changes included inviting physicians from Redemption Hospital to the CPD follow-up activity as a means of providing interprofessional development. None were able to join.

A barrier to spreading change was identified with the ProntoPacks. Disposable items had not been replaced by previous users of several of the ProntoPacks, and some of the non-disposable items, such as blood pressure machines, were missing. It was later determined that the non-disposable items were placed in other packs. A check of all ProntoPacks at the end of each training is necessary to ensure they are fully equipped for the next training. This will involve human and financial resources.

This follow-up CPD activity occurred during the pandemic. We experienced some problems with maintaining pandemic health protocols. Constant reminders were made to properly wear their masks, as social distancing is not possible if attention to fidelity to the healthcare setting and in this simulated clinical experience was to be maintained.

## Expanded Sustainability of the Normal Physiologic Birth CPD

### Spreading changes

Following the rollout of the CPD in February 2019, the LBNM and the World Continuing Education Alliance (WCEA) formed a partnership to develop an online portal for the delivery of continuous professional development learning for nurses and midwives. The WCEA mission is to improve access to education for health workers in lower to middle income countries. The portal offers users an app to download courses and study off-line and access to worldwide courses in a central place to record CPD credits. The system is linked to the LBNM files so will automatically update for re-licensure requirements.

Upon discussion between the LBNM, the U.S. academic institution, and the WCEA, it was agreed that the U.S. academic institution would rework the CPD content to create distinct modules, along with video of the two-day CPD event. The modular format now aligns with other CPD content on the LBNM portal. All content and video of the CPD went live by the end of December 2020. This will allow all midwives and nurses in Liberia to access this CPD at their own convenient time and pace.

## Discussion

The primary goal of this Continuing Professional Development Train-the-Trainer (CPD ToT) was to increase and expand the capacity of midwives and nurses to attend normal physiologic births with greater competence and confidence and also to support their knowledge and skills development in complex birthing situations, especial postpartum hemorrhage. Continuing professional development is an imperative in promoting quality and safety in the care of client populations [13].

This CPD ToT implemented a new regulatory requirement and pedagogical activity. A recent Liberian Board for Nursing and Midwifery policy requires that all future re-licensure applicants demonstrate the attainment of a set number of hours of CPD training. CPD accreditation involves completing necessary documents to outline CPD objectives and outcomes. This can be a time- consuming process.

Using simulation outside of traditional academic training was a novel approach in Liberia. Many of the participants have been in practice for some time so may not have had the experience of participating in simulation as students. Simulation involves the use of technology, planned scenarios, and pre- and debrief expertise. This involves both human and financial resources to be successful and consistent.

### Limitations and Lessons Learned

A clear and realistic timeline is necessary to implement any project. While all partners had an initially clear timeline, the contextual reality of long-distance communication, understanding regulatory and customary continuing professional development practices, and gaining access to stakeholders, facilities, and participants took longer than expected. Tuyisenge and colleagues [14] identified similar issues implementing their CPD in maternal health care in Rwanda. They identified the importance of conducting CPD trainings in health care facilities and regular retraining. Our CPD ToT was to happen in the largest public hospital in Liberia; however, financial resources prevented the training from occurring in this venue. The follow-up CPD activity did occur at the clinical facility.

The post-test data did not support a significant change in knowledge acquisition. While the CPD included simulation and active participation and demonstration by the attendees, there was no formal documentation of skills competency at the end of the training. The addition of an Observed Structured Clinical Examination (OSCE) would have added more robust evaluation evidence of the impact of the CPD training. We know that Liberia has one of the lowest literacy rates in Africa [15]. With this in mind, it is important that pre-test post-test scores not be the sole determinant of knowledge and skill gains.

Other lessons learned are supported by previous research on CPD implementation in low resource countries. System level challenges included funding and shortages of healthcare workers, which inhibits their ability to attend CPD trainings [16].

It is too soon since the completion of this CPD ToT to determine any resultant positive health outcomes. Anecdotal observations by participants and the clinical leaders suggest there have been some observed changes: a decline in complicated births, such as shoulder dystocia; a lower cesarian section rate; and an increase in completed partographs in the patient charts. While these were not intended outcomes of this CPD, the observation may demonstrate an increased awareness of maternal birthing outcomes from clinicians and leaders.

To see evidence of positive health outcomes, colleagues in Ghana spent many years and resources of their quality improvement project to support maternal and child health outcomes [17]. It is encouraging to note that this project used the same Model for Improvement [5], as did our project.

## Conclusion

Findings from this project highlight the complexity of designing and implementing a CPD Train-the-Trainer project in Liberia. While there have been many lessons learned over the course of this project, these lessons are an example of the importance of using a quality improvement framework for the development of future CPDs.

We have demonstrated the necessity of multiple partners to implement Liberia’s regulatory body’s recent policy for CPD development and approval. Using simulation requires expanded skill sets when implementing CPD trainings. Finally, including several assessments of knowledge, skills, and attitude acquisition are critical to determine the efficacy of any CPD.
